# Resequencing and Signatures of Selective Scans Point to Candidate Genetic Variants for Hair Length Traits in Long-Haired and Normal-Haired Tianzhu White Yak

**DOI:** 10.3389/fgene.2022.798076

**Published:** 2022-03-11

**Authors:** Qi Bao, Xiaoming Ma, Congjun Jia, Xiaoyun Wu, Yi Wu, Guangyao Meng, Pengjia Bao, Min Chu, Xian Guo, Chunnian Liang, Ping Yan

**Affiliations:** ^1^ Lanzhou Institute of Husbandry and Pharmaceutical Sciences, Chinese Academy of Agricultural Sciences, Lanzhou, China; ^2^ Key Laboratory of Yak Breeding Engineering, Lanzhou, China; ^3^ Guangdong Meizhou Vocational and Technical College, Meizhou, China

**Keywords:** yak, long-haired trait, DCMS, selection signal, resequencing

## Abstract

Tianzhu white yak is a rare local yak breed with a pure white coat in China. In recent years, breeders have discovered long-haired individuals characterized by long hair on the forehead in the Tianzhu white yak, and the length and density of the hair on these two parts of the body are higher than that of the normal Tianzhu white yak. To elucidate the genetic mechanism of hair length in Tianzhu white yak, we re-sequence the whole genome of long-haired Tianzhu White yak (LTWY) (*n* = 10) and normal Tianzhu White yak (NTWY) (*n* = 10). Then, fixation index (*F*_ST_), θπ ratio, cross-population composite likelihood ratio (XP-CLR), integrated haplotype score (iHS), cross-population extended haplotype homozygosity (XP-EHH), and one composite method, the de-correlated composite of multiple signals (DCMS) were performed to discover the loci and genes related to long-haired traits. Based on five single methods, we found two hotspots of 0.2 and 1.1 MB in length on chromosome 6, annotating two (*FGF5*, *CFAP299*) and four genes (*ATP8A1*, *SLC30A9*, *SHISA3*, *TMEM33*), respectively. Function enrichment analysis of genes in two hotspots revealed Ras signaling pathway, MAPK signaling pathway, PI3K-Akt signaling pathway, and Rap1 signaling pathway were involved in the process of hair length differences. Besides, the DCMS method further found that four genes (*ACOXL*, *PDPK1*, *MAGEL2*, *CDH1*) were associated with hair follicle development. Henceforth, our work provides novel genetic insights into the mechanisms of hair growth in the LTWY.

## Introduction

In taxonomy, yak (*Bos grunniens*) is a member of the *Artiodactyla*, family Bovidae, genus *Bos*, which is endemic to the alpine region of the Qinghai-Tibet Plateau (Qiu et al., 2015). Yak adapts to the cold climate and is distributed in China’s Qinghai-Tibet Plateau with an altitude of more than 3,000 m. The yak has been well known for its reputation as “boat on the plateau”, providing an indispensable transportation source for the production and life of local herdsmen (Qiu et al., 2015). Compared with cattle at a lower altitude, yak has long, thick skirt hair on the chest, legs, and flanks, forming a natural thermal insulation layer. Yak has more villi on their side, shoulder, and back, and the content of abdominal coarse hair (group hair) is the highest (Danzan et al., 2014).

Among all the 16 million yaks in the world, white individuals are rare, and the coat color is genetically unstable. Generally, in the domestic yak, most of the individuals’ coat colors are black, brown, black-brown, or with a small number of white patches, and only about 2–3% of the individuals are white (Wiener et al., 2003). The Tianzhu white yak population, however, is relatively large, with stable genetic properties, and is a unique local group breed in Tianzhu, Gansu province of China. As the special case of directional breeding for pure breeding in yak and the iconic white coat, Tianzhu white yak is a precious local yak group. In recent years, breeders have discovered a subgroup of Tianzhu white yak, which is characterized by the long hair on the forehead, and the length and density of the hair on the side of the body are higher than that of the normal Tianzhu white yak. We conducted statistical analysis on the production performance record and fur quality record data of Tianzhu White Yak in Tianzhu White Yak Breeding Base in Tianzhu County and concluded that Tianzhu White Yak can be divided into normal-haired type (≤13 cm) and long-haired type (>13 cm). Due to the economic benefit and landscape use of hair of Tianzhu white yak, breeders hope to breed stable offspring of this subgroup of Tianzhu white yak.

The selection signature of the genome includes the free-riding effect and selective clearance. The free-riding effect refers to that when a favorable mutation site with high fitness is fixed quickly, the polymorphism of the gene sequences linked to this site change accordingly (Smith and Haigh, 1974; Fay and Wu, 2000). Selective clearance is the phenomenon that the polymorphism of the chromosomal region linked closely around the site is reduced due to the free-riding effect (Smith and Haigh, 1974). And the selection in genetics often leads to corresponding changes in biological traits. These selected genes determine the traits of the organism. Therefore, it is equivalent to finding candidate genes that perform corresponding functions when the selection signals are identified. This is of great significance for understanding the evolutionary process of species and finding genes controlling traits with economic importance. Methods of the selection signal detection mainly include three categories, including population differentiation-based methods: fixation index (*F*_ST_) test (Pearse and Crandall, 2004), locus-specific branch lengths (LSBL) test (Shriver et al., 2004), and di test (Akey et al., 2010); allele frequency spectrum-based methods: Tajima’s D test (Tajima, 1989) and Hp test (Fay and Wu, 2000), etc.; haplotype-based methods: cross-population extended haplotype homozygosity (XP-EHH) test (Sabeti et al., 2007), extended haplotype homozygosity (EHH) test (Sabeti et al., 2002) and integrated haplotype score (iHS) test (Voight et al., 2006). In addition, the HapFLK method based on the hierarchical structure of the sample population and the cross-population composite likelihood ratio (XP-CLR) method based on the difference in multilocus allele frequency between two populations are always used in the selection signal detection (Hua et al., 2010; Fariello et al., 2013).

Multiple methods can be used to detect selection signals, and each method has its limitations. Results obtained from algorithms based on the genetic differentiation are interfered by the population history, and methods based on the unit points are affected by the linkage factors (Lewontin and Krakauer, 1973; Tajima, 1989; Shriver et al., 2004). In addition, methods based on the linkage-disequilibrium can only judge recent selection signals (Sabeti et al., 2002). Compared to the single-statistic tests or other meta-analyses, more recent studies manifested that composite measures of multiple signals selection have higher efficiency and present a reliable positional resolution (Grossman et al., 2010; Lotterhos et al., 2017). In our study, the de-correlated composite of multiple signals (DCMS), one of the composite analysis strategies was performed here (Ma et al., 2015). The DCMS method can combine *p*-values from different selection signal statistics into a single DCMS framework and correct for the overall correlation between the statistics based on the covariance matrix (Ma et al., 2015).

The purpose of this study is to identify the imprints left on the genome of LTWY and NTWY populations during the process of natural and artificial selection and to identify genes involved in the determination of hair length. To solve these problems, the genome-wide haplotype data of the long-haired Tianzhu white yak (LTWY) and the normal Tianzhu white yak (NTWY) were used, and five single methods (*F*_ST_, θπ ratio, XP-CLR, iHs, XP-EHH) and one composite method (DCMS) were conducted to detect the population selection signal and dig out the sites or candidate genes related to hair length where selection occurs. Our work provides an important reference for the selection and improvement of long-haired yak breeding.

## Materials and Methods

### Sample Collection and Sequencing

All blood samples of the LTWY and NTWY were collected from the Tianzhu white yak farmed in Gansu province, China. For each population, genomic DNA was extracted from blood samples using the EasyPure Blood Genomic DNA Kit (TransGen Biotech, Beijing, China) according to the manufacturer’s instructions. The quality and integrity of the extracted DNA were checked by measuring the A260/A280 ratio and screening by agarose gel electrophoresis. Qualified genomic DNA samples were randomly broken into fragments with a Covaris instrument. The interrupted samples were selected and concentrated around 200–300 bp using the Agencourt AMPure XP-Medium kit. The end-repair was performed on the fragmented DNA, the base A was added to the 3′ end to connect the sequencing adapter, and subsequent PCR amplification was performed on the ligated product. Then the PCR product was denatured to single-stranded, and the circularization reaction system was prepared, samples were mixed thoroughly and reacted at a suitable temperature for a certain time to obtain a single-stranded circular product. After digesting the linear DNA molecules that have not been circularized, the final libraries were obtained. The Agilent 2,100 Bioanalyzer (Agilent DNA 1000 Reagents) was used to detect the fragment size and concentration of the libraries, and then the qualified libraries were sequenced on the BGISEQ-500 platform. The raw image data obtained by sequencing were converted into raw reads by the BGISEQ-500 Base Calling software. The data were stored in the FASTQ file format.

### Reads Mapping and Single-Nucleotide Polymorphisms (SNPs) Calling

After removing adaptor sequences, contamination, and low-quality reads, high-quality reads were aligned to the latest *Bos grunniens* reference genome (accession number: GCA_005887515.1) using BWA-MEM (0.7.10-r789) with default parameters (Li and Durbin, 2009). The SAM files were sorted and converted to binary format (BAM, Binary sequence Alignment Map) to relieve computer memory and storage pressure via using SAMtools (version 1.9) (Li et al., 2009). The Genome Analysis ToolKit (GATK) (v4.1.8.0) was used to call variants. And alignments were marked for PCR duplicates using MarkDuplicates module of GATK following the BAM construction. For all the BAM files obtained, variants were called with HaplotypeCaller module. The g.vcf files were combined with the GenotypeGVCFs module of GATK. Finally, the original SNP files were obtained by using SelectVariants module (McKenna et al., 2010). With the VariantFiltration parameter, the filter conditions were set as “QUAL <30.0, QualByDepth (QD) < 2.0, Fisher`s exact test (FS) > 60.0, RMS Mapping Quality (MQ) < 40.0, HaplotypeScore >13.0”. After filtering, a VCF file containing high-quality SNPs was obtained. The command *samtools flagstat* was used to discover the statistic information of each sample, including average coverage, count of raw reads, mapped reads, and properly paired reads. The sequencing depth of each sample was analyzed using the VCFtools software. Considering that the low-quality genotype data may affect the subsequent analysis, samples were removed when individual call rate was <0.95 of all SNPs and SNPs with low call rate (geno<0.99), SNPs with low minor allele frequencies (MAFs) (MAF<0.05), SNPs without chromosomal assignments, and SNPs on sex chromosomes were excluded (Yurchenko et al., 2019). The parameter of PLINK was set as follow: -maf 0.05, --mind 0.05, --geno 0.01, and --chr 1–29.

### Principal Component Analysis (PCA)

Based on SNP information, PLINK was performed to the PCA to determine the genetic structure between populations. The visualization of PCA was based on the R package ggplot2.

### Selective Scans of Five Single Methods

Considering that method based on unit point SNP scanning is susceptible to factors such as genetic drift, therefore, a sliding window calculation strategy was selected here to raise the sensitivity of the selected signal and reduce false positives (Ma et al., 2015). The *F*_ST_ values were calculated using VCFtools software (v1.1.0) (Danecek et al., 2011) with parameter: -fst-window-size 50,000. Negative values of *F*_ST_ were converted into zeros. Nucleotide diversity (π) is the ratio of polymorphic sites in two randomly selected nucleotide sequences, which is evaluated on the difference between the sequences and the relative frequency (Tajima, 1983). In this study, the π values were also calculated by VCFtools software, and the parameter was set as -window-pi 50,000. The θπ ratio was calculated as π_L_/π_N_, where π_L_ and π_N_ were the nucleotide diversity values for the LTWY and NTWY, respectively. The values of the XP-CLR were calculated using the python script XPCLR, which was downloaded from GitHub (https://github.com/hardingnj/xpclr). The corresponding parameters were set as: maximum of SNPs 600, ld value 0.95, window size 50,000. The integrated haplotype score (iHS) was used to calculate values of a window of SNPs (Voight et al., 2006) through the R package rehh (Gautier and Vitalis, 2012). The software BEDtools was used to obtain the 50 kb window coordinate file (Quinlan and Hall, 2010). And the in-house python script was used to average absolute values of iHS into non-overlapping sliding windows of 50 kb. Lastly, the rehh package was also used for XP-EHH calculation (Sabeti et al., 2007). The NTWY population was selected as the reference population. And then, the same script was used to average the XP-EHH scores into non-overlapping sliding windows of 50 kb. To shorten computing time, the rehh package was performed in a parallel mode and the R package Parallel (Vera et al., 2008).

### De-Correlated Composite of Multiple Signals (DCMS)

After performing the statistics of five selection signal methods (*F*_ST_, θπ ratio, XP-CLR, iHS, XP-EHH), all the values were combined into a matrix based on the window name. The DCMS values were calculated using the MINOTAUR package (Verity et al., 2017). Firstly, the results of five statistics were converted to *p*-values using the function *stat_to_pvalue*. Each column in the input data frame was converted to fractional ranks between 0 and 1. These values were then transformed to rank-based *p*-values based on the one-tailed test (iHS- left-tailed; θπ ratio, XP-EHH, XP-CLR, and *F*_ST_–right-tailed). Final values were then transformed again to occupy the range 0–1 exclusive. Then, to obtain the correlations among these statistics, the covariance matrix was calculated using the *Cov-NAMcd* function with the parameters: alpha = 0.75, nsamp = 300,000. Combined with the matrix obtained in the last step, the *DCMS* function was used to calculate the DCMS scores. Robust estimations of the mean and variance of the DCMS scores were obtained using the R MASS package *rlm* function to eliminate the influence of isolated values (Boitard et al., 2016). And then the fitted DCMS scores were converted into *p*-values using the *pnorm* function. Finally, to control for false discovery rate, the R package *q*-value was used to adjust *p*-values using multiple hypothesis testing (Storey and Tibshirani, 2003). The adjusted *p*-values (*q*-values) were visualized by *manhattan* function of R package qqman (Turner, 2018).

### Variant Functional Annotations

Genes annotated in the BosGru3.0 genome version included in a selected interval were extracted using SnpEff (v4.5) software (Cingolani, 2012). The values of five selective signal methods in the top 1% of the empirical distribution (*F*_ST_>0.119, θπ ratio>2.558, |iHS|_LTWY_>1.532, XP-CLR>18.531, XP-EHH>2.203) were designated as candidate selection scans and genes that in those window region were defined as potential candidate genes. Next, to identify selection regions under the DCMS method of both populations, all the intervals with SNPs expressing decorrelated composite of multiple signal *q*-values less than 0.05 were obtained. BEDtools was used to extract the annotation file of these strong selection signal intervals. To get the meaningful mutations, the intron region and synonymous mutation sites were removed. The genes corresponding to the remaining sites were defined as potential candidate genes. The overlapping genes identified by DCMS were visualized using the circos package (Krzywinski et al., 2009). GO and KEGG enrichment was employed by KOBAS 3.0 (http://kobas.cbi.pku.edu.cn/index.php).

## Results

### Sequencing and Variation Calling

**FIGURE 1:**
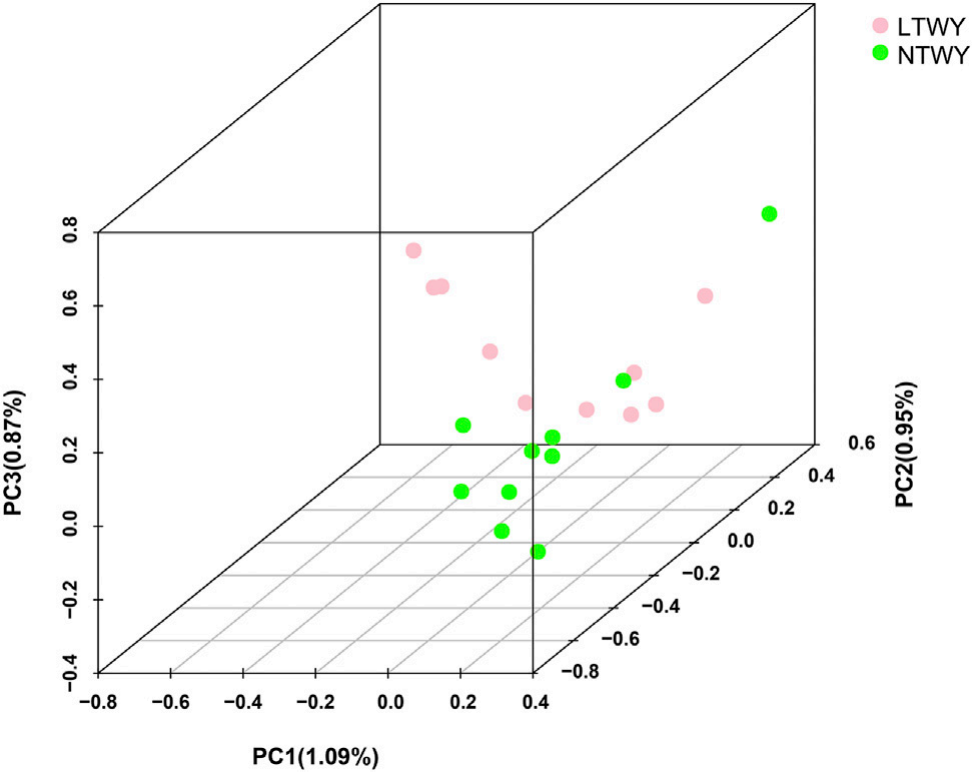
Principal component analysis of NTWY and LTWY populations.

In this study, a total of 20 samples from LTWY and NTWY were re-sequenced, and an average of 7.48× coverage was generated. High-quality reads were aligned using the LU_Bosgru_v3.0 reference genome through BWA MEM algorithm. Statistical results showed that a total of 3,014,148,570 reads were obtained, covering 98.33% of the reference sequences across the region (Table 1). The SnpEff software was used to evaluate the genomic polymorphism of the LTWY and NTWY populations (Table 2). Our results showed that a total of 15,124,083 SNPs were identified in the LTWY population, with an average of one mutation site per 169 bases on the chromosome. And a total of 15,331,905 SNPs were identified, with an average of one mutation site per 166 bases in the NTWY population. For downstream selective signal analysis, the g.vcf files of both populations were combined to identify SNPs, and finally, 16,708,655 SNP sites were obtained. In addition, the distribution region of SNPs in the LTWY and the NWTY was also analyzed (Table 2). Our results showed that the SNP variations in the LTWY and NTWY populations mainly occurred in the genetic interval (Intergenic), followed by the downstream interval (Downstream), upstream interval (Upstream), intron interval (Intron), exon interval (Exon) and so on (Table 2). And the Ts/Tv ratio which can be evaluated for the quality of the SNP call were 2.496 and 2.497 in the LTWY and NTWY, respectively. Principal component analysis results showed that two populations could be distinguished according to the three principal components. Three components captured 1.09, 0.95, and 0.87% of the total eigenvalue, respectively (Figure 1).

**TABLE 1:** Summary statistics of NTWY and LTWY re-sequenced reads.

Sample name	Number	Raw reads	Mapped reads	Properly paired reads	Average coverage	Average fold
NTWY	10	1,544,406,470	1,518,525,222	1,428,802,730	98.33%	7.58
LTWY	10	1,520,934,131	1,495,623,348	1,409,965,496	98.33%	7.38
Total	20	3,065,340,601	3,014,148,570	2,838,768,226	98.33%	7.48

**TABLE 2:** Functional annotation of the identified single-nucleotide polymorphisms (SNPs) in NTWY and LTWY.

Fields		NTWY	LTWY	Total
Sample counts		10	10	20
SNP count		15,331,905	15,124,083	16,708,655
Ts/Tv ratio		2.497	2.496	***
Hom/Het		0.61	0.63	***
SNP types				
Exon	Synonymous variant	128,679	132,764	152,356
	Initiator codon variant	28	23	16
	Start lost	243	271	208
	start_retained_variant	2	2	39
	Stop gained	1,840	1,780	1,857
	Stop lost	227	235	197
	Stop retained variant	130	123	190
Splice site	Splice region variant	25,046	25,556	26,836
	Splice acceptor variant	546	546	429
	Splice donor variant	724	725	629
Intron	Intron variant	13,948,749	13,869,588	15,421,077
	Intragenic variant	568	632	607
UTR	5 prime UTR variant	24,657	25,413	23,110
	5 prime UTR premature start codon gain variant	3,687	3,836	3,914
	3 prime UTR variant	55,993	57,992	60,922
Intergenic	Upstream gene variant	1,174,257	1,194,925	1,277,565
	Downstream gene variant	1,190,326	1,209,439	1,296,616
	Intergenic region	9,907,119	9,727,032	10,736,862
Functional classes	Missense	106,774	107,373	209,965
	Nonsense	1,840	1,780	3,746
	Silent	128,813	132,891	252,509

### Analysis Results of Single Selection Signal Method

**FIGURE 2:**
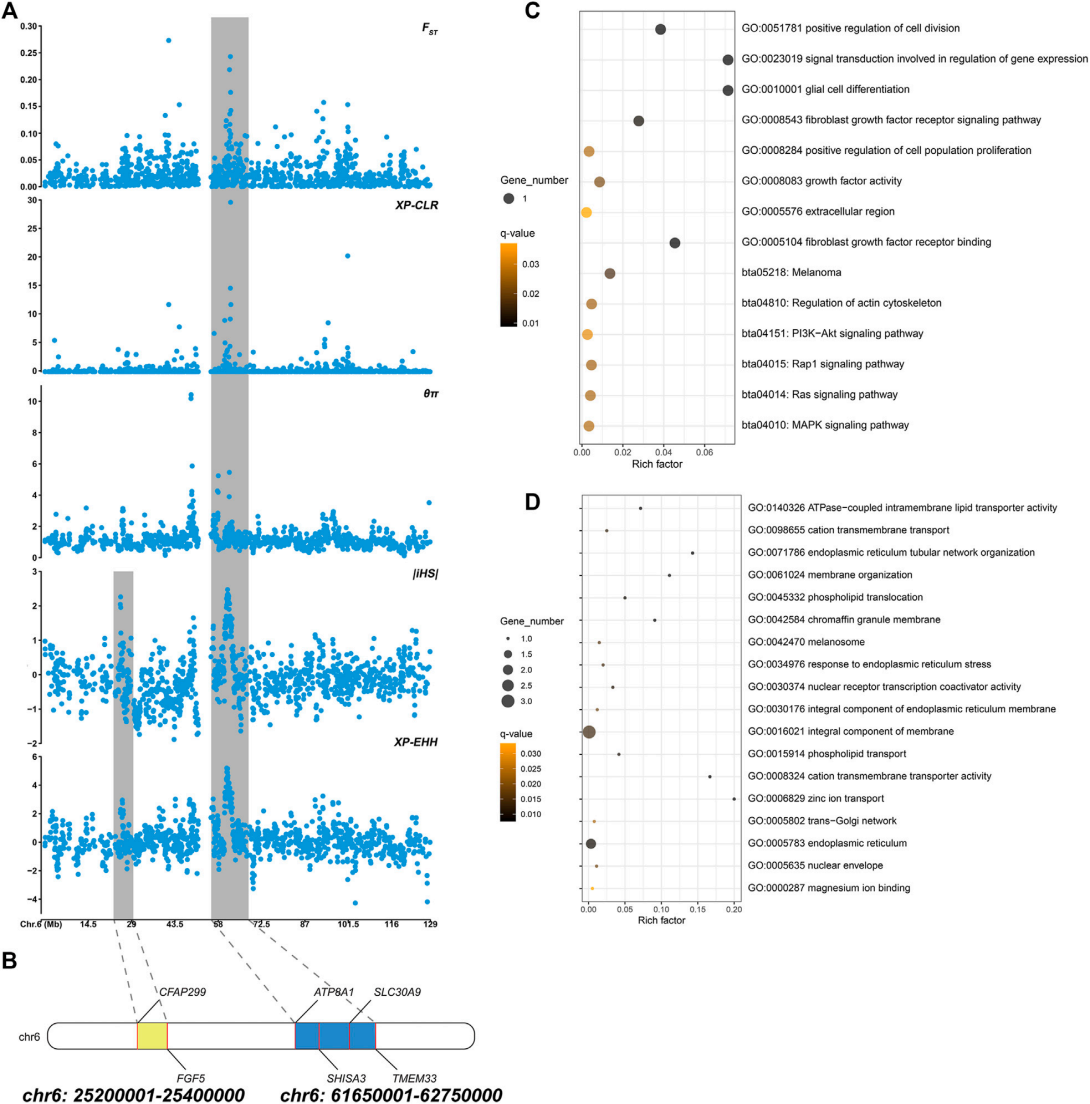
**(A)** Manhattan plot of five selection signals on chromosome 6. The two hotspots are marked with shading. **(B)** Genes identified in two hotspots. **(C)** Functional enrichment analysis of 0.2-MB hotspot. **(D)** Functional enrichment analysis of 1.1-MB hotspot.

Based on different principles, five selection signal methods were used to screen selected regions and candidate genes. By two haplotype-based selection methods (iHS, XP-EHH), a hotspot (chr6: 25,200,001-25,400,000) with a length of 0.2-MB was detected (Figure 2A). Part of the segment of two genes (*FGF5*, *CFAP299*) was located in this region (Figure 2B). One missense mutation (c.302G > C, p.Ser101Thr) sites with large allele frequency differences were identified in *FGF5* (Table 3). Enrichment analysis results showed that two genes in the 0.2-MB hotspot were involved in eight GO items and six KEGG pathways, including signal transduction involved in the regulation of gene expression, fibroblast growth factor receptor binding, growth factor activity, positive regulation of cell population proliferation. The KEGG pathway includes MAPK signaling pathway, PI3K-Akt signaling pathway, Rap1 signaling pathway, Ras signaling pathway, melanoma, regulation of actin cytoskeleton (Figure 2C). Among the selected areas identified by the five methods, we found another hotspot (chr6: 61,650,001-62,750,000) with a length of 1.1-MB (Figure 2A). Four genes (*ATP8A1*, *SHISA3*, *SLC30A9*, *TMEM33*) were annotated in this hotspot (Figure 2B). In *SHISA3*, we identified one missense mutation (c.199G > A, p.Ala67Thr) sites (Table 3). Enrichment analysis results showed that four genes in 1.1-MB hotspot were enriched in 18 GO items, including magnesium ion binding, trans-Golgi network, cation transmembrane transport, membrane organization, phospholipid transport, etc (Figure 2D). There was no significant KEGG pathway enrichment in this segment.

**TABLE 3:** Allele frequencies for missense mutations in the candidate genes identified in NTWY and LTWY.

Sites	Gene	Amino acid variation	Allele frequency (NTWY)		Allele frequency (LTWY)		Genotype	Genotype frequency (NTWY)	Genotype frequency (LTWY)
			Before mutation	After mutation	Before mutation	After mutation			
c.302G > C	*FGF5*	Ser101Thr	1.00	0.00	0.78	0.22	CC	1.00	0.67
							CG	0.00	0.22
							GG	0.00	0.11
c.199G > A	*SHISA3*	Ala67Thr	0.90	0.10	1.00	0.00	CC	0.90	1.00
							CT	0.00	0.00
							TT	0.10	0.00
c.958G > A	*ACOXL*	Asp320Asn	0.95	0.05	1.00	0.00	CC	0.90	1.00
							CT	0.10	0.00
							TT	0.00	0.00
c.1360G > T	*CDH1*	Val454Leu	1.00	0.00	0.85	0.15	CC	1.00	0.80
							CA	0.00	0.10
							AA	0.00	0.10
c.2274C > A	*MAGEL2*	His758Gln	0.00	1.00	0.06	0.94	GG	0.00	0.00
							GT	0.00	0.11
							TT	1.00	0.89
c.325A > G	*MAGEL2*	Met109Val	0.86	0.14	1.00	0.00	TT	0.90	1.00
							TC	0.00	0.00
							CC	0.10	0.00
c.1579T > C	*PDPK1*	Ser527Pro	0.95	0.05	1.00	0.00	AA	0.90	1.00
							AG	0.10	0.00
							GG	0.00	0.00

### Analysis of the Multi-Signal De-correlation Composite

**FIGURE 3:**
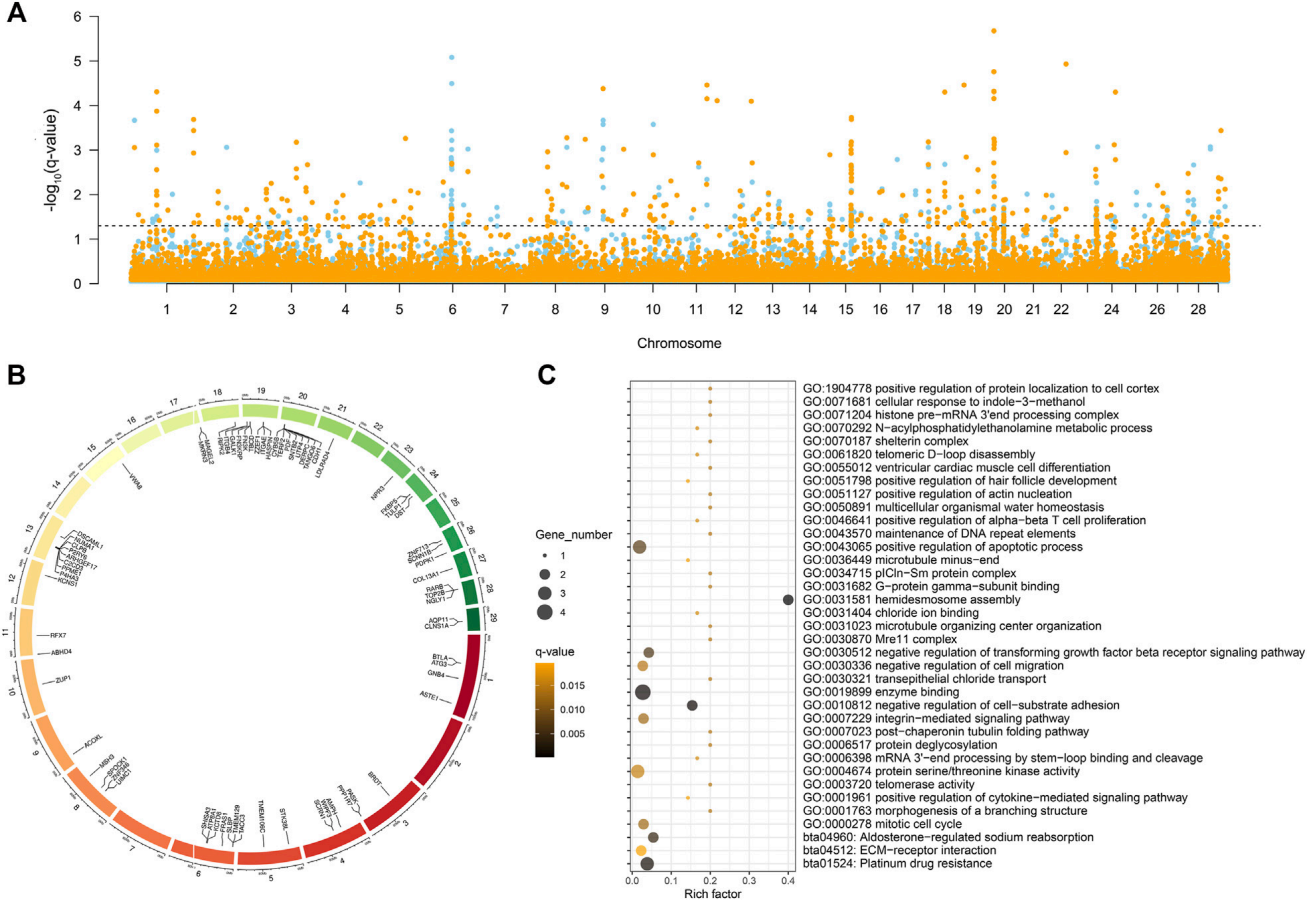
**(A)** Manhattan plot of decorrelated composite of multiple signals *q*-values of NTWY (skyblue) and LTWY (yellow) populations. **(B)** Overlapping genes identified by DCMS method between NTWY and LTWY. **(C)** Functional enrichment analysis of overlapping genes.

The five selection signal statistics were combined into a single DCMS framework using the MINOTAUR. 400 and 420 genomic intervals under putative selection in LTWY and NTWY genomes were obtained after fitting for normal distribution, calculation of *p*-values, and correction for multiple testing (*q*-value < 0.05) (Figure 3A). According to the DCMS method, we screened the loci in the overlapping region for annotation. A total of 254 intervals were obtained, and 71 genes were annotated (Figure 3B). GO enrichment analysis resulted in 34 significantly enriched pathways (*q*-value < 0.05). Hair follicle development, including positive regulation of hair follicle development, positive regulation of cytokine-mediated signaling pathway, mitotic cell cycle, positive regulation of apoptotic process, negative regulation of transforming growth factor beta receptor signaling pathway, and negative regulation of cell-substrate adhesion were involved as major enrichment pathway, which may play an important role in the hair growth of LTWY. In addition, three significant pathways (platinum drug resistance, aldosterone-regulated sodium reabsorption, ECM-receptor interaction) were enriched in these genes (Figure 3C).

The DCMS method also identified strong signals detected near the 1.1-MB hotspot, further verifying the feasibility of this method. Further screening identified some highly significant genes related to hair follicle development. Among all the overlapping genes, we noticed that both LTWY and NTWY populations had a common signature of selection near the *ACOXL*, *PDPK1*, *MAGEL2*, and *CDH1* (Figure 3B). *ACOXL* encoded the rate-limiting enzyme of the fatty acid β-oxidation pathway (Schrader and Fahimi, 2006). One missense mutation (c.958G > A, p.Asp320Asn) site was found in this gene. In the 50 kb window (20:10300001-10350000), one missense mutation site (c.1360G > T, p.Val454Leu) of *CDH1* was identified (Table 3). *CDH1* played an important role in maintaining the adhesive properties and proper skin differentiation in keratinocytes (Hodivala and Watt, 1994). Two missense mutations (c.2274C > A, p.His758Gin; c.325A > G, p.Met109Val) were identified in *MAGEL2* in the 50 kb window (17: 76450001-76500000) (Table 3). *MAGEL2* was primarily expressed in the paraventricular nucleus, supraoptic nucleus, and in the suprachiasmatic nucleus (SCN) of the hypothalamus, which played role in circadian rhythm (Panda et al., 2002). *PDPK1* was associated with glucose metabolism (Beg et al., 2017), and one missense mutation (c.1579T > C, p.er527Pro) was found (Table 3).

## Discussion

The resequencing data from LTWK and NTWK was used to reveal the potential selective sweeps. And detailed genomic information along with candidate genes associated with the phenotypic change in the long-haired population was identified here. Our results showed that the total number and the distribution density of SNPs, Ts/Tn, and heterozygosity of the two yak populations were close (Table 2), indicating that the genetic diversity of the two yak populations was similarity. The PCA results showed that the degree of differentiation between the two populations was low, suggesting that two population had a closer relatedness (Figure 1). Tianzhu white yak is a local breed that has been bred artificially for a long time. Previous studies showed that the Tianzhu white yak has a large variation within the population. The long-haired type should be a subgroup that appeared in a short period, and the degree of differentiation from the normal type of white yak is lower (Qiu et al., 2015). The PCA result was consistent with the current population situation of long-haired white yak.

To reveal the genetic mechanisms of the long-haired phenotype, five selection (*F*_ST_, XP-EHH, iHS, XP-CLR, θπ ratio) methods were performed to find candidate genes and pathways. Window scan results showed a shared strong selection region of 0.2 MB in length (CHR6:25,200,001-25,400,000) was detected on two haplotype-based selection methods, and two genes (*FGF5*, *CFAP299*) were located (Figure 2B). Currently, *FGF5* is the famous mutant gene found in mammalian species that causes the hairy phenotype variation. Long hair is inherited as a simple recessive trait in animals (Drögemüller et al., 2010). Studies on long-haired mice, dogs, rabbits, and donkeys have shown that the inherited hair length is caused by mutations within *FGF5* gene (Drögemüller et al., 2010; Dierks et al., 2013; Legrand et al., 2014; Zhao et al., 2018). In addition, these genes were enriched into four hair follicle-related pathways (Ras signaling pathway, MAPK signaling pathway, PI3K-Akt signaling pathway, and Rap1 signaling pathway) (Figure 2C). Ras signaling is essential for skin development (Drosten et al., 2014). He et al. found Ras and Rap1 signaling pathways were involved in the growth of hair follicle stem cells cultured *in vitro* (He et al., 2020). The MAPK signaling pathway can induce the proliferation and differentiation of hair follicle cells, promote the periodic development of hair follicles, and then affect the growth of villi and the distribution of hair follicles and the number of hair shafts (Zhang et al., 2008; Öztürk et al., 2015; Lu et al., 2021). PI3K/Akt signaling pathway is essential for *de novo* hair follicle regeneration (Chen et al., 2020). Previous studies have found that the PI3K/AKT and ERK1/2 signaling pathways in hair follicle cells can work together to accelerate the transformation of hair follicles from resting phase to growth phase, extend the growth phase of hair follicles, and promote hair follicle development and hair growth (Liu et al., 2020). In addition, a 1.1-MB hotspot (CHR6:61,650,001-62,750,000) was found in five selection signals, and four genes (*ATP8A1*, *SHISA3*, *SLC30A9*, *TMEM33*) were annotated (Figure 2B). *SLC30A9* and *ATP8A1* were involved in several pathways related to ion transport, including cation transmembrane transport, magnesium ion binding, zinc ion transport, and cation transmembrane transporter activity pathway (Figure 2D). Hair development is closely related to the content of various ions, such as zinc, which plays an important role in animal hair growth (Vallee and Falchuk, 1993). Suliman et al. (1988) found that their wool was sparse and their growth rate slowed down, and the wool fell off on both sides of the back and neck when sheep were zinc deficient (Suliman et al., 1988). Zinc deficiency was also leading to rough fur and shedding in cattle (Ott et al., 1965; Tomlinson et al., 2004). *TMEM33*, *SHISA3*, and *SLC30A9* were also enriched in phospholipid translocation, phospholipid transport, trans-Golgi network, response to endoplasmic reticulum stress (Figure 2D). These pathways were involved in the synthesis of extracellular proteins, which related to may be related to the synthesis of hair growth-related proteins (Shore and Tata, 1977; Vitale et al., 1993). In summary, these genes may affect hair growth through ion transport or the synthesis of extracellular proteins.

Due to the low degree of differentiation between the two populations, the method based on genetic differentiation may not be able to identify different genes. A combination of several selection methods may be more conducive to this research, and the DCMS method allows more precisely and filters out spurious results specific to other methods (Ma et al., 2015). We calculated DCMS statistics for each population and the overlapping genes were selected as candidate genes associated with phenotypes (Figure 3A). In our study, a total of 71 overlapping genes were obtained using the DCMS method (Figure 3B). These overlapping genes were enriched into pathways involved in hair follicle development, including positive regulation of hair follicle development, positive regulation of cytokine-mediated signaling pathway, mitotic cell cycle, positive regulation of apoptotic process, negative regulation of transforming growth factor beta receptor signaling pathway, negative regulation of cell-substrate adhesion (Figure 3C). Classic studies showed that during embryogenesis, the embryonic *epidermis* and mesenchyme communicated with each other to form a hair follicle (McElwee and Hoffmann, 2000). The strong selection signal of DCMS found on Chromosome 20 (20:10300001-10350000) contained the *CDH1* gene, which mediated the intercellular adhesion in the mammalian *epidermis* and hair follicles as the adhesive component of adherens junctions (Hodivala and Watt, 1994). *CDH1* was weakly expressed in the dermis, while was highly expressed in the *epidermis* and hair follicles (She et al., 2016). Reports showed that *CDH1* played an important role in the formation of melanin in hair follicles and the adhesion of hair follicles and *epidermis* (Larue et al., 1994; Perl et al., 1998; Young et al., 2003; Kuphal and Bosserhoff, 2012). Previous studies also found that continuous hair follicle cycling was dependent on *CDH1* (Young et al., 2003). *ACOXL*, a typical lipid metabolism-related gene, was strongly selected in our study (Figure 3B). This enzyme could catalyze the desaturation of acyl-CoAs to 2-trans-enoyl-CoAs in the reductive half-reaction (Brown and Baker, 2003). Festa et al. (2011) found that dermal white adipose tissue (WAT) not only provided animals with thermo-insulation but also modulated regeneration dynamics of pelage hair follicles via the production of paracrine growth factor. Regeneration of the dermal WAT periodically cycles was in synchrony with the hair cycle, undergoing the cycles of expansion and collapse (Chase et al., 1953; Donati et al., 2014). These pieces of evidence suggested that the lipogenesis and lipolysis of WAT could be influenced by the β-oxidation process, so we inferred that *ACOXL* may affect the metabolism of WAT to synchrony affect the hair follicle cycle in yak. One circadian rhythm-related gene (*MAGEL2*) was identified among the overlapping genes (Figure 3B). *MAGEL2* has been found to modulate the circadian rhythm: it was primarily expressed in the suprachiasmatic nucleus where the transcription of *MAGEL2* oscillated in phase with clock-controlled genes. In addition to local paracrine modulators, hair follicles are also regulated by physiological changes that take place throughout the body. For example, several results suggested the involvement of the circadian clock regulate the hair cycle and hair follicle pigmentation (Al-Nuaimi et al., 2014; Hardman et al., 2015). In the mature anagen, clock genes were prominently expressed in the hair matrix, dermal papilla, and other follicular compartments (Plikus et al., 2013). Previous research showed that mice deficient in *Magel2* expression will disrupt circadian rhythm, metabolic and endocrine deficits (Kozlov et al., 2007; Mercer et al., 2013). Therefore, *MAGEL2* may affect the hair growth cycle by influencing the robust rhythmicity of *MAGEL2* expression, which may be one of the reasons for the different hair lengths of Tianzhu white yak. *PDPK1* was involved in the negative regulation of the transforming growth factor beta receptor (TGF-β) signaling pathway. During the development process of the hair follicle, TGF-β1, TGF-β2, and their receptors were locationally and cyclically specifically expressed in hair follicles and were proved to be involved in regulating the growth and development of hair follicle through multiple signaling pathways. Studies of transgene or gene knockout of TGF-β also confirmed that TGF-β related signaling was necessary for hair follicle development. It is indicated that PDPK1 may play an important role in hair development and cycle through TGF-β (Paus et al., 1997; Foitzik et al., 1999).

Through five selection signal methods (*F*_ST_, XP-EHH, iHS, XP-CLR, θπ ratio), 0.2-MB and 1.1-MB hotspot were identified, both located on chromosome 6. *FGF5* was identified as the key gene affecting hair length in 0.2-MB hotspot. The enriched pathways (Ras signaling pathway, MAPK signaling pathway, PI3K-Akt signaling pathway, and Rap1 signaling pathway) were involved in the process of hair length differences. The genes (*ATP8A1*, *SHISA3*, *SLC30A9*, *TMEM33*) annotated in 1.1-MB hotspot mainly enriched into two types of pathways, one was ion transport-related pathways, another was endoplasmic reticulum related pathways, which may affect hair follicle development through protein synthesis. The DMCS method further obtained four genes related to hair follicle development (*ACOXL*, *PDPK1*, *MAGEL2*, *CDH1*), which influenced the hair follicle cycle through fat metabolism, growth factors, circadian rhythm, and cell adhesion pathways. The candidate genes and pathways screened in this study were involved in the formation mechanism of hair length in yak. In the next step, further experiments will be performed to verify the function of candidate genes. Our study provided an important reference for breeding, breed improvement, and functional genome research of landscape Tianzhu White yak in China.

## Data Availability

The bioproject number of the sequencing data information about long-haired Tianzhu white yak and normal-haired Tianzhu white yak is PRJNA766811 in the NCBI Sequence Read Archive.
